# Creating equity in health research to drive more and better evidence

**DOI:** 10.12688/wellcomeopenres.17287.1

**Published:** 2022-01-18

**Authors:** Trudie Lang, John Reeder, Vasee Moorthy, Nísia Trindade Lima, Glenda Gray, Pontiano Kaleebu, Chikwe Ihekweazu, Sabin Nsanzimana, Gagandeep Kang, Michael Makanga, Soumya Swaminathan

**Affiliations:** 1 Centre for Tropical Medicine and Global Health, Nuffield Department of Medicine, University of Oxford, Oxford, OX3 7FZ, UK; 2 Department of Research for Health, World Health Organization, Geneva, Switzerland; 3 Special Programme for Research and Training in Tropical Diseases (TDR), World Health Organization, Geneva, Switzerland; 4 The Oswaldo Cruz Foundation (FIOCRUZ), Rio de Janeiro, Brazil; 5 South African Medical Research Council, Western Cape, South Africa; 6 Medical Research Council/Uganda Virus Research Institute and London School of Hygiene and Tropical Medicine Uganda Research Unit, Entebbe, Uganda; 7 Nigeria Centre for Disease Control (NCDC), Federal Ministry of Health, Abuja, Nigeria; 8 Rwanda Biomedical Centre (RBC), Kigali, Rwanda; 9 The Wellcome Trust Research Laboratory, Division of Gastrointestinal Sciences, Christian Medical College, Vellore, India; 10 European & Developing Countries Clinical Trials Partnership (EDCTP), The Hague, The Netherlands; 11 World Health Organization, Geneva, Switzerland

**Keywords:** health research, research framework, inequity in health research, LMIC, equity in health research

## Abstract

Health research is rapidly changing with evidence being gathered through new agile methods. This evolution is critical but must be globally equitable so the poorest nations do not lose out. We must harness this change to better tackle the daily burden of diseases that affect the most impoverished populations and bring research capabilities to every corner of the world so that rapid and fair responses to new pathogen are possible; anywhere they appear.

We must seize this opportunity to make research easier, better and more equitable. Currently too many nations are unable to generate the evidence or translate it to directly change health outcomes in their own communities. It is essential to act and harness this emerging change in how research data can be generated and shared, so that all nations sustainably gain from this development. There are positive examples to draw on from COVID-19, but we now need to act. Here we present an initiative to develop a new framework that can guide researchers in the design and execution of their studies. This highly agile system will work by adapting to risk and complexity in any given study, whilst generating quality, safe and ethical data.

## Disclaimer

The views expressed in this article are those of the author(s). Publication in Wellcome Open Research does not imply endorsement by Wellcome.

## The inequity in who benefits from Health Research

We need to support an increase in the quality, volume and diversity of research across all nations and epidemiological settings to improve outcomes through better surveillance and diagnostics, risk factor determination, new prevention, treatment and management strategies and better understanding of the genetic, social and economic drivers of poor health in all settings. One of the fundamental barriers to healthcare practitioners undertaking research is the view that research is something remote and not for them ^1^. It is perceived as too difficult or too expensive with a common opinion that getting involved with ‘research’ is about working on clinical trials to evaluate a new regulatory product, and that is something only senior doctors do ^1, 2^. However, research should be an integral component function of healthcare delivery and be seen as ‘do-able’ by all actors within hospitals, laboratories, clinics and community health centres. Some health research can be highly pragmatic; about measuring what you see, evaluating new, or improved, interventions and processes and using evidence within decision-making.

Whilst 90% of preventable deaths do occur in resource limited countries, only less than 10% of health research is undertaken in these regions. For example, sub-Saharan Africa contributes only less than 1% of biomedical publications globally; this is not static, with slowing improvements but still far behind ^3^. Unfortunately, a common situation still occurs where impactful studies are conducted by residents of resource limited countries on behalf of western organisation, who then publish results in high impact journals that ironically inaccessible to the same resource limited countries where the data originated ^4– 6^.

We believe in finding new ways to enable research to be applicable to every setting. Disease outbreaks, such as Ebola, Lassa fever, Zika and now COVID-19 highlight that all types of health research data are equally important ^7– 11^, from epidemiological, clinical, social science to intervention studies and implementation research, and that research needs to be guided and supported so that it is always scientifically sound, safe, ethical and accurate ^10, 12, 13^.

Zika was a good case to consider; because of the paucity of our knowledge, epidemiological data was critical in to the understanding of the scale and situation, sampling studies were required to understand the disease morphology, and social science to explain the context and perceptions, all required to guide acceptable study designs and possible interventions. Clinical trials were not undertaken within the Zika outbreak, as there were no products to test. Had there been a vaccine however, all these data would have been vital to the design and execution of a successful regulatory trial programme. This story has repeated with COVID-19, where we needed rapid, quality data to understand the virus and characterise the disease in order to determine its clinical impact and find potential preventive and therapeutic interventions, and design the right trials on the right individuals.

Outbreaks are a clear example of why the ability to undertake health research is needed in all countries. Research must be done firstly in the originating country to address the unknowns in those first crucially important cases and then everywhere there is transmission so that data is captured during that all-important unknown window where there are enough cases to answer the questions.

The WHO Research and Development (R&D) Blueprint was put in place after the Ebola crisis to address these gaps by enabling cross-cutting preparedness for research in epidemics. This has made strong impact within the COVID-19 pandemic with studies being coordinated, promoted and supported across the globe, including in many LMICs. The generation of core protocols, standard outcome measures, a global safety review committees and ensuring data sharing ^14^ has all enabled the generation of faster, high quality evidence.

The pandemic has shaken the globe and created such devastating impact. Meanwhile, day-to-day diseases of course remain, and still need to be tackled. It is also important to understand the interaction between communicable and non-communicable diseases (NCDs). NCDs are becoming the commonest causes of death and disability in developing countries, yet WHO’s Global Observatory on Health R&D highlights the marked lack of research focusing on NCDs in these settings; gaps include product formulations tailored to developing country needs and research on risk factors, how to deliver NCD screening and management in resource poor settings. The same applies to research needs for injury prevention in developing countries. Looking across Sustainable Development Goal (SDG) 3 it is difficult to find SDG targets that would not benefit from targeted research to increase progress development.

Alongside this universal need for more and better research, we are on the edge of a transformation in how research is undertaken ^15, 16^. Digital health records are a significant driver in this; alongside adaptive design approaches and international recognition, there needs to be some fundamental changes to enable clinical trials to be less administratively restrictive, unduly expensive and cumbersome ^12, 17– 19^. It is predicted that within a decade we will no longer be undertaking research in the stop-start and highly fragmented way that it is now performed ^16, 20^. Currently, we move in disjointed steps from epidemiology to observational sampling studies and into clinical trials, then differentiation into phase I, II, III and IV clinical trials for the evaluation of investigational products. However, this is with the exclusion of non-phased evaluation of diagnostics and prognostics, where we too need better and faster research approaches. We have been working to this step-wise process for over 50 years; our funding systems, guidelines and regulations are set-up around these norms, largely having been developed around product development using paper-based technology. The changes already happening consider research in a wider sense than just trials, in that the whole ecosystem of health research is now recognised.

Whatever design or approach used, all data should be collected to high quality, ethics and safety standards and all should be shared, in order that others could take part and contribute or run their own studies using these informative datasets. Such an agile and adaptive approach must be designed to be both ethically and regulatory compliant, working from the best available information to ensure the safety of participants while ensuring that important investigations are continued and the social context is considered. Study designs could be better and safer because real-time data monitoring can be more responsive, both to assess, safety and to determine whether a research question has been adequately answered.

Any activity which is beyond standard care and where information is being collected as data for research requires fully informed consent, carefully considered and designed around the risk, complexity and nature of the study. Therefore, a protocol and all the necessary review, approvals and regulations also then become a necessity. If the specific questions set within the protocol could pull data from health records, rather than having separate databases it would increase efficiency, standardisation and enable faster progress, and more information on the participant would be available. This is not so far away, and indeed happening already in terms of studies that analyse real world data ^16, 20^. Adaptive design is a commonplace and it is considered a given that all studies will extract data from health records in the future. This evolution could transform our ability to understand diseases and find better ways to tackle the burdens that they bring much faster than is currently possible. Indeed, this evolution from collecting data specifically for research, towards the collection of research data being an embedded element of healthcare would create a more fluid, learning environment. Phase I and challenge trials create different challenges and there are other situations where research questions would set the situations apart from participant records, or that patient records may not exist. The point is the need for agility and approaches designed to the question and clinical, social and epidemiological setting, all focusing on the goal of safe, ethical, accurate and cost effective, rational research.

Surely we need to be ready for this change? And if so, we have some progress to make, since currently our stop-start design, regulatory, review and funding processes are not fully geared to work outside of the old norms of phase I-IV clinical trials, even though that shift is already happening in some parts of the world ^16, 20^. If we miss this opportunity we risk impeding progress by having systems in place that stifle rather than facilitate better research to tackle health challenges. Rather than being late recipients in these advances, countries with the greatest burdens on health should be ahead of this curve and be part of this evolution in generating new processes and guidance to encourage good research and make sure all data is collected accurately, safely and ethically. While researchers may be ready and willing in resource-limited nations to move forward with changing how research is designed and done, medical research is not easily trusted, particularly when there is industry engagement. Building trust is not simple, but preparing for and ensuring discussions and a strategy to work with political leadership, media and society is essential to make sure that all nations gain from this progress, and resource limited nations (those in fact who have the most to gain), are not left behind.

## What is Health Research?

We know that addressing this first fundamental question will support more research, as currently there is confusion over what is research and what is an audit, or public health implementation. We know that the perceived difficulties of stepping over the ‘line’ into undertaking ‘research’ is preventing many healthcare providers from engaging in research in the first instance.

Here we suggest a viable definition of research that works for any capture of data where informed consent would be required, and therefore a protocol and ethical approval are ensured. This is the point where the caregiver becomes a researcher; and so, this point needs to be easily defined. We think this definition is practical and clear. It would be useful to have a globally accepted definition and as such, we present this for comment:

**Health Research is the assessment of biomedical or health-related outcomes that are either observational or interventional with and where the intention of collecting these data is to derive generalisable new knowledge.**

## Creating parity

As a decisive issue in bringing health research closer to the needs of the populations of developing countries and regions, it is important to have a greater democratisation in the generation of scientific knowledge in these countries. One should not accept a situation in which a few countries and research groups direct global research on health even with merit and social commitment. There is a pressing need for research networks to be more symmetrical and equitable in defining problems, objects, research protocols and their translation to the society. Strengthening and reducing the global imbalance in health research is a prerequisite for narrowing the gap between knowledge and the needs of the people and territories in which they live. To this end, it is necessary to support the entry of the developing countries in precision public health that uses health knowledge in the digital age for the social needs, leaving no regions or no people behind. Having a research base, industrial production and high-density knowledge services is vital to the equity and democratisation of health research.

To be ahead of these changes we need a universal research-enabling framework, by which research teams could be guided in the design and operational delivery of their study. A highly agile, adaptable and easy to use system would allow teams to design and deliver studies that would collect quality data, protect the safety of the participants and be conducted to high scientific and ethical standards. All of which would encourage the delivery of more and better, rational and high-quality health research.

Such a framework needs to align appropriately with the declaration of Helsinki ^13^, and be easy to apply to all types of research. It is not true to say that only clinical trials need research guidelines, such as International Committee for Harmonization - Good Clinical Practice ICH-GCP. All types of study risk inaccurate data, unethical processes, or procedures that could cause harm. It is important to have quality, reliable data from all types of studies, if we are to generate evidence to improve health outcomes and, equally important, to mitigate harm from all these studies in all disease areas. There should not be different standards, whether research is being conducted for commercial goals, product registration or specific diseases or locations. We need a highly adaptable framework that is designed to work for all research.

This framework would provide guidance relative to the risk and complexity of a specific study and would guide relevant, appropriate and proportionate application of regulations and guidelines, such as ICH-GCP, where needed. However, this would work to assure the accuracy, safety and ethics of all types of study, which are the basic principles behind ICH-GCP but difficult to apply to other types of research, which this would solve. In addition, it would contain new elements, such as community engagement, good participatory practice and assuring quality, safety and ethical standards within qualitative data. Health research studies can involve the giving of an intervention, sample-taking or qualitative elements, or any combination, and this framework should guide the accuracy, safety and ethics for operations and data capture in any or all of these.

## WHO; Taking the lead and working with others

The World Health Organization (WHO) have a new focus on embedding research into healthcare ^11^. Research is transitioning fast in terms of new adaptive designs, fluidity of study types and using digital health records. We think that an accessible and highly adaptable research framework could be developed relatively easily. This tool could guide researchers in setting up high-quality health research studies in context, where the design and conduct will mitigate, or identify and then resolve, any potential risks to the study reliability, data quality, patient safety and ethical standards.

There is a need to be ready for, or even better, be ahead of this change. This is important, as this is an opportunity to harness this evolution and ensure it brings global benefit and increases the collection of better and faster evidence to drive change. Doing nothing is risking widening the inequity gap between those experiencing benefits from health research and those missing the opportunity. We already see areas such as oncology and cardiology leading the way with these changes; it is important that low-resourced regions, neglected diseases and vulnerable populations benefit from these changes too.

The WHO intend to act to address this gap and will be working with a wide body of partners to develop this new framework for health research. The overall goal is to drive the capture of more and better health research data within every healthcare setting across the globe that can bring benefit to all.

## Data availability

No data are associated with this article.
