# Supplementary material for: Creating equity in health research to drive more and better evidence
Source: Wellcome Open Res. 2022 Jan 18;7:15. [Version 1] doi: 10.12688/wellcomeopenres.17287.1 (PMC10685064; doi:10.12688/wellcomeopenres.17287.1)
Supplement: Peer review history

# Reviewer report: Reviewer response for version 1

**DOI:** 10.21956/wellcomeopenres.19112.r68278

**Published:** 2023-11-28

**Authors:** Roni M Ellington

**Affiliations:** 1 Michigan State University College of Human Medicine, East Lansing, Michigan, USA

In this open letter, the authors provide a rationale for developing a research framework that can be used to inform equitable and responsive health research. Specifically, the authors discuss how the current practices used to collect, analyze and distribute health research and its findings are inefficient, inaccessible, inequitable, and lack a comprehensive framework that could be used to guide research. In addition to arguing for the need for a research framework, the authors provide a compelling rationale for using digital health records as a potential data source for research, which would allow clinical trails to to be "less administratively restrictive, unduly expensive and cumbersome." Finally, the authors discuss work engaged by the WHO to develop protocols, standard outcome measures and a global safety review that articulates the  need to develop consistent protocols for equitable and accessible global research. 

 Overall, the authors provide a solid rationale for the need to develop a research framework and protocols to inform global research and provides compelling support for the claims made in the article, specifically for the need to expand the equitable and ethical use of digital health records to support better and more timely health research. This definition I found concise, clear and consistent with the current understandings of research in health settings.

 Although the argument for the need for a comprehensive research  framework, protocols, expanded use of digital health records and more equitable access to research is inherently compelling, I am not sure the contribution that this open letter will have in the development of such a framework and subsequent protocols. There is little discussion of a draft of this framework nor is there a clear discussion of the key dimensions of this emerging framework. What would have made it a stronger piece is by providing some details of the emerging framework and using the rationale provided as a introduction to the emerging health research framework. Also, there is little discussion as to how this framework should be developed and by whom and whether or not the framework would address the issues raised to support its development. It is implied that a framework would  address the issues of inefficiency, lack of equity, and forwarding a global standard for health research (I suppose using digital health records, however I was not clear of the connection between the two based on my reading of the document), it is unclear how it would do so and whether or not this can be achieved given some of the challenges discussed in the article. It seems as if the authors spend much of their time articulating the need for equity and a new framework, they do not provide a process through which this can be done equitably and make a case for how this framework would be developed. This seems like an important component of the open letter that has not be discussed adequately.

 In addition, there is an implication that using digital health records for the purpose of research would assist in timely and equitable global research. However, it is not well argued as to how these digital health records could be used to foster research or how they would fit into the overall framework proposed. This may not be doable given the focus and purpose of health records and how this can lead to many other ethical issues that are not discussed in the article. 

 There needs to some thoughtful discussion as to how data collected for the purpose of individual's health could be ethically used in research given that the authors themselves point out the current challenges of getting data for research purpose ( since much of these challenges are generally put in place to mitigate unethical uses of data). 

 I found the writing of the article someone confusing and requires editing. I would recommend that the authors get this piece edited for clarity, word choice and flow so that is would read better to the audience. Also the words research and data were used interchangeably in the article and they are not synonymous. Also there are paragraphs for which the main idea is not clear and the organization of the paragraph fails to support the main idea of the paragraph. This makes the piece hard to read and cumbersome. Editing the document for clarity would help make the argument clear and coherent.

 Overall, I think the authors do a solid job of arguing for a framework to inform global health research; however, the authors fail to make a compelling case for how this should be done, who would be involved, how the framework would foster efficiency and equity in health research, and what would be some key elements of the framework that would address the issues raised in the article. Further, the use of digital health records for research is compelling and has potential to streamline heath research, the ethical and technical issues inherent in this suggestion must be teased out to make for a compelling case for not only the use of these data but how they should be integrated in this research framework and protocols

Does the article adequately reference differing views and opinions?

Partly

Are all factual statements correct, and are statements and arguments made adequately supported by citations?

Yes

Is the Open Letter written in accessible language?

Partly

Where applicable, are recommendations and next steps explained clearly for others to follow?

Yes

Is the rationale for the Open Letter provided in sufficient detail?

Yes

Reviewer Expertise:

Evaluation and Health Equity research

I confirm that I have read this submission and believe that I have an appropriate level of expertise to confirm that it is of an acceptable scientific standard, however I have significant reservations, as outlined above.

# Reviewer report: Reviewer response for version 1

**DOI:** 10.21956/wellcomeopenres.19112.r68275

**Published:** 2023-11-02

**Authors:** Robin L Pierce

**Affiliations:** 1 University of Exeter, Exeter, England, UK

The Open Letter by Prof Lang and colleagues highlights the need for systematic improvement in research–enabling practices for evidence generation globally. They ground this goal in a call for a framework that supports equitable participation in health research that is at once responsive and forward looking. This timely call is rooted, in part, in a recognition that without deliberate and dedicated attention to the pervasive inequities in health research, including in capacity, funding, and publication and career progression opportunities, the logical trajectory will be the widening of the inequity gap, particularly in terms of benefits gained and responsiveness of research to global needs and contours. This call for a research–enabling framework that serves both short term and long term needs and goals carries with it multiple challenges attendant to the various dimensions of what such a framework must do, how it should be applied to achieve designated goals, and by what process such a framework should be developed. While it is central to clarify and foreground the core objectives of the call, it is also necessary to consider collateral effects, structural limitations, and the possibility of enhanced risks. The Open Letter considers some of these and presumably sees them as surmountable. This is a bold call in that it invites the development of a new way of doing things that centres equity (now and in the future) and requires the global research community to systematically build capacities accordingly. While stressing the essentiality of equity, the authors identify the need for “a highly adaptable framework that is designed to work for all research”, specifically challenging current norms regarding clinical trials, including their centrality in health research as well as the systems that favour the already well established.

 There are many critical aspects of a call for an equity creating framework, three of which are 1) requirements of a research–enabling framework for equity in health research 2) the need to accompany the pursuit of broader benefits with careful regard for the emergence of known and novel risks and the need for the development of appropriate protections and 3) the process of framework development and implementation.

 1. A research–enabling framework

 A core motivation for this call for an equity-grounded, research-enabling framework is the need for research to be more responsive globally. Lang and colleagues refer to examples that highlight the rapid response needed in cases of disease outbreak as well as the relative paucity of research on non–communicable diseases (NCD) that occupy an increasing share of the burden of disease in LMIC. Thus, the proposed framework must address issues of symmetry between the incidence, types, and burden of disease and response capacity, enabling timely response to public health needs wherever they arise. To do this the framework must facilitate the capacity to execute research in a variety of settings around the world such that deployment of effective response does not rely on the presence and expertise of foreign researchers for the necessary research to be conducted. Lang and colleagues appreciate the complexity of achieving this goal, acknowledging challenges at the local, national, regional, and international levels. Such a framework must be future – regarding as well as indicate what must be done today to build and maintain capacity in the short and long term. As the letter acknowledges, there are many challenges of and dimensions to building sustainable capacity, including patterns of hierarchy in the conduct of research in LMIC, (over–) reliance on clinical trials as the most valued form of evidence ^1^, funding patterns and trajectories, as well as the absence of career incentives ^2^. As Prof. Lang and colleagues have noted, questions regarding selection of research targets (data collection) and other possibly outcome–affecting phenomena require input by local researchers. Identifying the reasons for the current inequity gap in health services research in LMIC, why 90% of research helps less than 10% of the global population ^1^, and what will make a difference in increasing capacity in various LMIC countries, for example, are integral to the successful development of a framework.

 Given the centrality of equity in the call for a framework, it seems important to stress the multilayered nature of the underlying causes of inequity ^1^ and, therefore, the nature of interventions that will be needed to ensure that the framework can achieve what it is developed to do. Many questions will need to be addressed in order to get at the multifactorial causes of inequity. For example, to what extent will the framework be designed to accommodate or address structural inequities? How should the framework approach the enduring inequitable effects of past hierarchies on research skills and capacity both within local teams as well as between foreign and local partners?

 2. Pursuit of benefit through more and better evidence

 Prof. Lang and colleagues emphasize the need for more and better evidence and refer to different types of evidence that could enhance research, ultimately serving to yield better outcomes. The authors suggest that a new framework could systematically facilitate not only new research practices but also new research targets. Responsive to local knowledge of issues, challenges, barriers, intersecting practices, incorporating additional types of data could be significant to crafting effective interventions in the short and long term as well as in crisis situations. As such, the framework that Lang and colleagues call will shift practices to facilitate the collection of data on a range of potentially outcome–affecting phenomena that may be solely within the gaze of local teams but are currently neither collected nor acknowledged as possibly impactful. Underscoring this, the authors assert that clinical trials may not be the optimal form of evidence generation ^1^, and certainly not in all situations and for all health issues. They specifically point to the value of embedding data collection into healthcare delivery. The potential significance of electronic health records and other digital forms of measuring, storing, and generating health information cannot be ignored in the development of the proposed framework. The increased recognition of the value of real-world evidence offers another example of rapid evidence generation needed to respond to crisis. Nevertheless, the monitoring and surveillance that these practices require is not insignificant. Along with the benefits of introducing or expanding the use of these modes of data collection, increased, altered, or novel risks must be considered. That is, with new forms or types of data collection, there will be a need to ensure that protections track these data processing practices. This requires, among other things, consideration of the risks *locally* for data breaches affecting vulnerable members of populations. In the case of embedding data collection in healthcare delivery, we much query whether data collection of sensitive information will have an adverse effect on healthcare seeking in certain cultural environments. The emergence and pervasiveness of digital technologies forming some component of health research deserves attention in this framework, not least because of the inequitable uptake or access of digital technologies and the potential resulting impact on health research and outcomes. The increasingly important need for adequate cybersecurity measures worldwide should obviously also be observed in the context of research in LMIC. Dedicated attention to protections against known, emerging, and novel risks including and beyond data protection and privacy, will need to accompany this progressive step toward creating equity in health research. It will be essential to acknowledge the changing risk landscape brought about by implementation of the framework and facilitate the development of effective protections.

 3. Process

 The Open Letter makes several critical observations about the need for a framework to move toward equitable evidence generation globally. This initiative that aims to create equity in health research through a research–enabling framework comes at a critical time in which timely response to public health needs is essential, with impacts often felt worldwide if not adequately dealt with locally. Nevertheless, the development of such a framework may be as much about *process* as about *outcomes*. While we urgently need to prioritize and centre equity in health research, the process by which this is done is likely to be critical to its success. Lang and colleagues rightly suggest that the framework will contain “new elements” in research” such as participatory research. But it may be worth considering which elements may be integral to the process of developing the framework. How and to what extent participatory elements should be adopted in the development of the framework itself. If part of the goal is to develop new guidelines in recognition of the new types and modes of research, then the various levels of impact on local persons and communities will form important input into the framework, for example. Once developed, how will the framework adapt and conform to new insights, knowledge, and evidence? In some ways, it would seem to necessarily be an evolving document in order to meet the task that the authors have assigned to it. The authors could offer some early thoughts on the nature of the framework as a “living” document.

 This call for an equity-creating framework is quite ambitious. Thinking through the multiple types and levels of changes that such a framework would necessarily encourage, a critical piece will be how such a framework would be evaluated and assessed. And, if necessary, revised to reflect new insights. Just as researchers and oversight bodies came to recognise the need for a revision of the longstanding Helsinki Declaration ^3^, various developments, including the rapid evolution and introduction of technologies used to conduct research point to a reality that consists of the presentation of continually new challenges. Procedures for amendments, updates, and significant revision could be helpful to timely response to unanticipated challenges.

 Additionally, there is substantial complexity in widening the scope data collection in terms of cultural, legal, and other norms. Explicit consideration of the implications of expanding data collection and what would be needed to ensure responsible data collection should accompany the pursuit of the benefits of more data. Moreover, an increase in the scope of data collection may require consideration of infrastructural capacity to provide adequate protections, particularly given the increasingly critical role of cybersecurity.

 Finally, the role of process seems fundamental given the motivation, objectives, rationale of the call for such a framework. Some have argued that process is integral to the pursuit of equity ^4^. The role of process can rightly be expected to occupy a significant place in the development of this framework. Even before its development, highlighting the role of process could be valuable in grounding this ambitious work in its equity objectives. The call for a framework for creating equity in health research is both timely and urgent as the impact of the Covid pandemic has made clear. Recognition by the World Health Organisation and others of the broad need for equity in health research is an important step toward bringing this call forward.

Does the article adequately reference differing views and opinions?

Partly

Are all factual statements correct, and are statements and arguments made adequately supported by citations?

Yes

Is the Open Letter written in accessible language?

Yes

Where applicable, are recommendations and next steps explained clearly for others to follow?

Yes

Is the rationale for the Open Letter provided in sufficient detail?

Yes

Reviewer Expertise:

Health Policy, Health Technology Innovation, Regulation of Technology, Research Ethics, Translational Digital Health, AI in Healthcare

I confirm that I have read this submission and believe that I have an appropriate level of expertise to confirm that it is of an acceptable scientific standard.

## References

[1] : Plug COVID-19 research gaps in detection, prevention and care. *Nature* .2020;583(7816) : 10.1038/d41586-020-02004-1 333 10.1038/d41586-020-02004-1 32669692

[2] : Strategies for developing sustainable health research capacity in low and middle-income countries: a prospective, qualitative study investigating the barriers and enablers to locally led clinical trial conduct in Ethiopia, Cameroon and Sri Lanka. *BMJ Open* .2017;7(10) : 10.1136/bmjopen-2017-017246 e017246 10.1136/bmjopen-2017-017246 29030412PMC5652508

[3] : World Medical Association Declaration of Helsinki. Ethical principles for medical research involving human subjects. *Bull World Health Organ* .2001;79(4) :373-4 11357217PMC2566407

[4] : Caregiving as moral experience. *Lancet* .2012;380(9853) : 10.1016/s0140-6736(12)61870-4 1550-1 10.1016/s0140-6736(12)61870-4 23130363

# Reviewer report: Reviewer response for version 1

**DOI:** 10.21956/wellcomeopenres.19112.r68281

**Published:** 2023-11-01

**Authors:** Arish Mudra Rakshasa-Loots

**Affiliations:** 1 The University of Edinburgh, Edinburgh, Scotland, UK

This Open Letter summarises issues of inequity in health research, highlights the ongoing transformation of health research by the integration of digital health records, and makes the case for a universal framework to guide research design (presumably for research involving digital health data) for all researchers, but especially to bridge the gap for clinicians aiming to carry out health research in LMICs.

 In my opinion, the greatest contribution made by this Open Letter is the proposed definition of "health research", which I found to be concise and widely applicable.

 Beyond this definition, I struggle to identify precisely the contribution made by this Open Letter. The authors make a compelling case for developing a universal research framework to guide all health-related research and provide some features that they think such a framework should have. I think developing such a framework is an ambitious (but important) undertaking, even though the authors suggest that it could be achieved “relatively easily”. However, this article does not in fact describe a developed (or even in-progress) version of this framework. As such, I wonder if this Open Letter would not have been more suitable, in a more condensed form, to serve as the background and rationale for this framework *once it has been developed* and is being shared with the research community, rather than as a stand-alone piece referring to a hypothetical framework which *may* be developed and shared in future.

 I also find there is a misalignment between the emphasis on creating equity in this Open Letter and the purpose of this proposed framework. As it stands, the title and abstract of the Open Letter suggest that this framework will be specifically focused on creating equity, with no specific reference to digital health data. Upon closer inspection, I understood this framework to guide all health-related research, with a focus on research which involves digital health data, thereby *implicitly* creating greater equity between researchers in the Global North and the Global South. I would recommend re-working the title and abstract of this Open Letter at least to clarify that the purpose of this framework is to guide the design and delivery of research anywhere in the world which involves digital health data, and this framework may in turn contribute to greater equity in health research. (Unless I have misunderstood the purpose of the framework – see below.)

 There are two specific statements in the article which require further support for the authors’ arguments. First, the authors say “it is considered a given that all studies will extract data from health records in the future”. I am not convinced that this is a given. Accessing health records may not be relevant or appropriate for all health-related research. Could the authors either provide some citations for this claim, or present a more detailed argument? Second, the authors argue that “There should not be different standards, whether research is being conducted for commercial goals, product registration or specific diseases or locations.” Again, I am not convinced that this is true. While I appreciate the authors’ case for a universal research framework which can guide researchers anywhere in the world and set a global standard for health research, I am unsure whether a “grand unified framework” for health research – highly adaptable though it may be – can truly replace the need for different levels of regulatory and ethical considerations for research depending on the anticipated outcomes. Could the authors clarify their phrasing, or present a more detailed argument as to why they believe that a single research framework could/should replace more tailored research guidelines for different types of health research?

 Beyond these substantive critiques, I believe this article requires comprehensive editing. As a researcher from the Global South and second-language English speaker myself, I am deeply apprehensive about suggesting this (as critique of language is unfortunately all too common in peer reviews, and often driven by bias). However, I found that the issues in writing in several places of the article actively hinder the reader’s understanding of the authors’ arguments, and therefore need to be rectified. While I won’t provide a comprehensive list of editing suggestions, some observations are noted below:

- Some extremely long sentences which are very difficult to follow (see, for instance, the first sentence of the main text).
- Some unclear phrasing (e.g. “so that data is captured during that all-important unknown window where there are enough cases to answer the questions” / “direct global research on health even with merit and social commitment” – unclear what these phrases mean).
- Some overly general sentences, which do not necessarily contribute to the authors’ arguments and detract from the academic tone of the article (e.g. “The pandemic has shaken the globe and created such devastating impact. Meanwhile, day-to-day diseases of course remain, and still need to be tackled.”)
- Some contradictory phrasing (e.g. “Strengthening and reducing the global imbalance in health research”).

Does the article adequately reference differing views and opinions?

Partly

Are all factual statements correct, and are statements and arguments made adequately supported by citations?

Partly

Is the Open Letter written in accessible language?

Partly

Where applicable, are recommendations and next steps explained clearly for others to follow?

Partly

Is the rationale for the Open Letter provided in sufficient detail?

Partly

Reviewer Expertise:

HIV mental health

I confirm that I have read this submission and believe that I have an appropriate level of expertise to confirm that it is of an acceptable scientific standard, however I have significant reservations, as outlined above.
